# Reviewed article: Freitas AKMSO, Camargo JDAS, Souza ATB, Pontes THA, Oliveira JMP, Vieira LA, Ferreira PLM, Camargo JF, Freitas JCOC. Sociodemographic and clinical factors associated with delayed diagnosis of cervical cancer: a cross-sectional study, Brazil, 2016-2020. Epidemiol Serv Saude. 2025:34;e20240764

**DOI:** 10.1590/S2237-96222025v34e20240764.b

**Published:** 2025-09-08

**Authors:** Arn Migowski

**Affiliations:** 1 Instituto Nacional de Câncer, Rio de Janeiro, RJ, Brazil

## Editor’s review

## First round comments

Prezados autores,

Na conclusão do resumo, consta que “Os resultados destacam a urgência de políticas públicas para melhorar o acesso e a detecção precoce.”. Ficou indefinido do que se trata ao acesso. Confirmação diagnóstica? Tratamento? E o “melhorar a detecção precoce” também pode se referir a um problema de acesso ou qualidade, entre outros. Ao mesmo tempo é preciso cautela para não sair muito do que os dados do estudo suportam.

Será preciso ajustar a frase “Nos Estados Unidos, análises ajustadas para incidências específicas na população histerectomizada revelaram que, entre 2000 e 2012, mulheres negras apresentaram uma taxa de mortalidade ajustada por idade mais de duas vezes superior à de seus pares brancos (23). Na referência original, que é um editorial, é descrita “merged national databases, adjusted for population-specific incidences of hysterectomy,”. O artigo original esclarece que foram estratificados de forma equivalente sobre a prevalência de histerectomia para mulheres de 20 anos ou mais de forma a remover mulheres que não estavam em risco do denominador. “Seus pares brancos” também precisa ser traduzido corretamente.

Recommendation: Minor revision

